# Phytosterol Profiling of Apiaceae Family Seeds Spices Using GC-MS

**DOI:** 10.3390/foods10102378

**Published:** 2021-10-08

**Authors:** Ramesh Kumar Saini, Min-Ho Song, Ji-Woo Yu, Xiaomin Shang, Young-Soo Keum

**Affiliations:** 1 Department of Crop Science, Konkuk University, Seoul 05029, Korea; saini1997@konkuk.ac.kr (R.K.S.); hlhkkl@konkuk.ac.kr (M.-H.S.); wooody96@konkuk.ac.kr (J.-W.Y.); 2 Jilin Provincial Key Laboratory of Nutrition and Functional Food, Jilin University, Changchun 130062, China; xmshang@jlu.edu.cn

**Keywords:** β-sitosterol, stigmasterol, caraway (*Carum carvi* L.), fennel (*Foeniculum vulgare* Mill.), celery (*Apium graveolens* L.), anise (*Pimpinella anisum* L.)

## Abstract

Phytosterols are nutritionally vital phytoconstituent owing to their cholesterol (low-density plasma lipoprotein-cholesterol, LDL-C)-lowering, anti-inflammatory, and antioxidant properties. Among the widely used spices and herbs, the seeds spices of the Apiaceae family represented the healthiest fatty acid profile. Thus, to explore the other health-beneficial lipids, the present study was aimed to analyze the phytosterol profile of eight seed spices of the Apiaceae family, utilizing gas chromatography (GC)-mass spectrometry (MS). The sterols contents calculated on an oil (mg/100 g of oil) and spice weight (mg/100 g spices; dry weight) basis varied significantly among the seed spices (*p* < 0.05; Turkey HSD). The β-sitosterol and stigmasterol were the most dominating sterols among the studied spices, together accounted for 40.3 (Ajwain) to 69.8% (celery) of total sterols in the seed oil. Among the studied spices, the oil extracted from caraway seeds showed the highest total sterols (602.2 mg/100 g of oil). Interestingly, based on spice weight, fennel seeds also showed the similar highest number of total sterols (134.2 mg/100 g in fennel and 133.3 mg/100 g in caraway), owing to the high contents of oil (25.9%) in fennel seeds. Overall, celery, caraway, fennel, and anise seeds oil are rich sources of health-beneficial phytosterols.

## 1. Introduction

Spices are a vital part of human nutrition worldwide [1]. Spices are food adjuncts traditionally used as coloring, seasoning, flavoring, and food preservative agents [1,2]. Moreover, spices are a rich source of nutritionally important phenolic compounds [3] responsible for antioxidant, anti-inflammatory, hypolipidemic, antibacterial, antiviral, anticancer, and digestive stimulative properties [4,5,6,7,8].

The Apiaceae family plants are well known for their richness in essential oils and nutritionally vital compounds, phenolic compounds, and petroselinic acid (C18:1n-12) [9]. In our recent investigation, among the several spices and herbs, cumin, coriander, fennel, and dill and seeds spices of the Apiaceae family represented the healthiest fatty acid profile, based upon fat quality indices such as the atherogenic index (AI), and the thrombogenic index (TI), and the ratio of hypocholesterolemic/hypercholesterolemic (h/H) fatty acids [10].

Spices are not a significant source of sterols, fatty acids, and other nutrients, as they form a small part of the diet. However, among the widely used spices and herbs, the seeds spices of the Apiaceae family represented the healthiest fatty acid profile [10]. Despite the vast popularity of dill (*Anethum graveolens* L.), celery (*Apium graveolens* L.), and Ajwain (*Trachyspermum ammi* L.), the phytosterol composition data are not available. Phytosterols are well known to reduce low-density plasma lipoprotein-cholesterol (LDL-C) levels [11,12,13] and thereby lower cardiovascular risk. According to an estimate, a daily intake of 3 g plant sterols/stanols reduced serum LDL-C concentrations by 12% [12]. Moreover, as an antioxidant, plant sterols, such as campesterol, β-sitosterol, stigmasterol, and scavenge the harmful reactive oxygen spices and thus prevent lipid peroxidation [14]. Furthermore, animal and human studies have demonstrated the anti-inflammatory effects of phytosterols [15]. Considering these facts, although spices form a small part of the diet, consuming phytosterol-rich spices may improve the health benefits of the diet.

Moreover, among the previous studies, a significant variation exists in the sterol composition of Apiaceae family seeds spices. For instance, in oil extracted from coriander (*Coriandrum sativum* L.) seeds, β-sitosterol contents are reported from 100.9 mg/100 g [16] to 231.4 mg/100 g [17]. Similarly, in oil extracted from anise (*Pimpinella anisum* L.) seeds, β-sitosterol contents are reported from 211.6 mg/100g [18] to 626.4 mg/100 g [16]. Moreover, recently, Balbino et al. [18] recorded a significant amount of α-spinasterol (206.2 mg/100 g oil) in anise seed oil, while it was not detected in previous studies [16,19].

Furthermore, many studies reported the sterol composition of Apiaceae family seeds utilizing a gas chromatography (GC)-flame ionization detection (FID) based analysis method [16,19,20,21], which may yield a misidentification of compounds eluting at a similar retention time. This problem can be eliminated in the gas chromatography (GC)-mass spectrometry (MS) based method, as the fragment ions of analytes are also compared with the standards.

In view of the above-listed facts, a detailed and comparative phytosterols profile of seeds spices of the Apiaceae family may be helpful to identify those with health-beneficial potential. Thus, the present study was aimed to analyze the phytosterol profile of eight most widely used seed spices of the Apiaceae family, namely dill (*Anethum graveolens* L.), celery (*Apium graveolens* L.), caraway (*Carum carvi* L.), coriander (*Coriandrum sativum* L.), cumin (*Cuminum cyminum* L.), fennel (*Foeniculum vulgare* Mill.), anise (*Pimpinella anisum* L.), and Ajwain (carom; *Trachyspermum ammi* L.) utilizing a recently validated GC-MS method [22].

## 2. Materials and Methods

### 2.1. Plant Material, Reagents, and Standards

A total of eight commercially packed seed spices (200–500 g each spice from at least three different brands) of the Apiaceae family, namely dill (*Anethum graveolens* L.), celery (*Apium graveolens* L.), caraway (*Carum carvi* L.), coriander (*Coriandrum sativum* L.), cumin (*Cuminum cyminum* L.), fennel (*Foeniculum vulgare* Mill.), anise (*Pimpinella anisum* L.), and Ajwain (carom; *Trachyspermum ammi* L.) (Appendix A) were obtained from retail outlets in Seoul, Korea. The spice samples of different brands were mixed in equal proportions (200–300 g total) to make a representative sample, ground into a fine powder using a 7010HG laboratory blender (Waring Commercial, Torrington, CT, USA), placed into an air-tight container, and stored at room temperature.

The standard sterol compounds, campesterol, stigmasterol, β-sitosterol, α-spinasterol, and epicoprosterol (internal standard) were obtained from Merck Ltd., Seoul, Korea. The organic solvents used for the extraction of lipids were of high-pressure liquid chromatography (HPLC) grade, obtained from Samchun Chemical Co., Ltd., Seoul, Korea.

### 2.2. Extraction of Sterols

Crude lipids containing sterols were extracted from grounded seeds spices following the recently optimized protocol [10,22], with minor modifications based on the previous report [23]. The butylated hydroxytoluene (BHT: *w*/*v*; synthetic antioxidant; Merck Ltd., Seoul, Korea) was added to the extraction solvent (0.75%, *w*/*v*) to minimize the degradation of sterols [24]. The total crude lipids were hydrolyzed [23], silylated utilizing *N*,*O*-bis(trimethylsilyl)trifluoroacetamide (BSTFA) containing 1% trimethylchlorosilane (TMCS), and analyzed by GC-MS. A detailed extraction, hydrolysis, and silylation procedure is illustrated in Appendix B.

### 2.3. GC-MS Analysis of Sterols

Sterols were analyzed after silylation with trimethylsiloxy groups [TMS; −O-Si(CH_3_)_3_] utilizing QP2010 SE GC-MS equipped with a fused silica Rxi-5ms column (30 m, 0.5 μm film thickness, 0.25 mm ID; Restek Corporation, Bellefonte, PA, USA). Helium was used as a carrier gas maintained at the pressure control flow of 62.1 kPa (7.8 mL/min total flow).

The injector and MS ion source were precisely maintained at 275 °C, while the MS interface was maintained at 280 °C. The column oven temperature was kept at 120 °C for 1 min, then progressively increased to 300 °C with the linear increase of 15 °C/min, and held at 300 °C for 27 min [22]. The 1 µL of samples and standards were injected utilizing an autosampler. For the identity confirmation, retention time and fragmentation pattern were compared with authentic standards and reference databases (NISTO8S, NIST08, and Wiley9). The sterols were quantified using the six-point calibration curve (25–400 µg/mL) of standard sterols.

The GC-MS method used for the quantification of sterols was validated (in terms of accuracy, linearity, precision, and stability) in our recent study [22]. The limit of detection (LOD) of quantified sterols assessed based on the standard deviation of the GC-MS response and the slope [25] was recorded above the 8.14 µg/mL, while and limits of quantitation (LOQ; 3.3 × LOD) was above the 24.42 µg/mL. Recoveries of sterols were precisely monitored and normalized using epicoprosterol (5β-cholestan-3α-ol) as internal standards.

### 2.4. Statistical Analysis

A total of six replicate extraction and analysis were performed for each representative spices sample. The results were analyzed using IBM SPSS statistics (version 25) employing a one-way analysis of variance (ANOVA), considering a significance level of 0.05 (Turkey HSD).

## 3. Results and Discussion

### 3.1. Identification of Sterols by GC-MS

**Figure 1:**
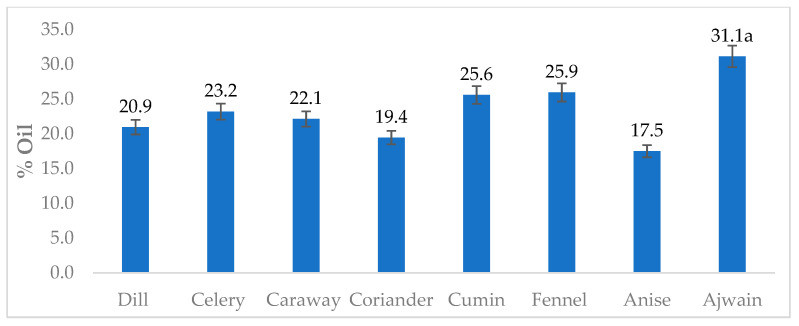
The oil contents (%, dry weight basis) recorded in the Apiaceae family spices. The values are mean ± standard deviation, from an average of six determinations. ^a^ The mean value is significantly highest among the studied spices (*p* < 0.05; Turkey HSD).

**Figure 2:**
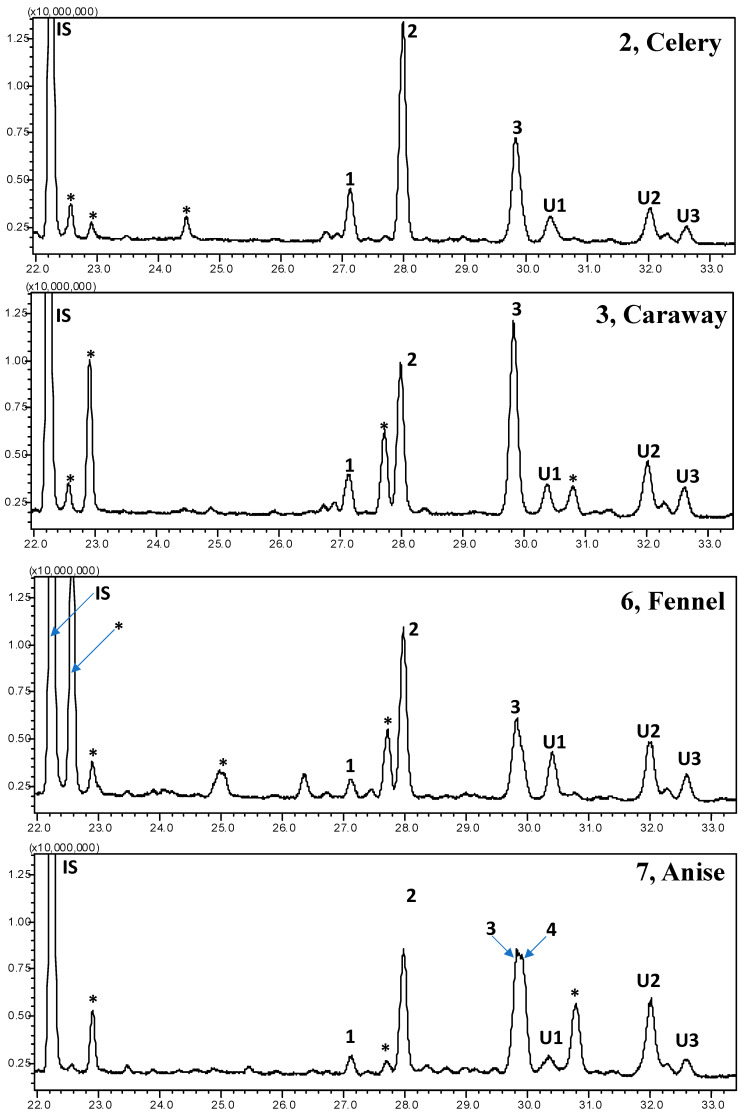
The gas chromatography (GC)- total ion chromatograms (TICs) of sterols identified from celery, caraway, fennel, and anise seeds (seed oil). The peak numbers are as follows: (1) campesterol, (2) stigmasterol, (3) β-sitosterol, and (4) α-spinasterol. IS: internal standard (epicoprosterol; 5β-cholestan-3α-ol; CAS 516-92-7); U: unidentified; * non-sterols compounds.

**Figure 3:**
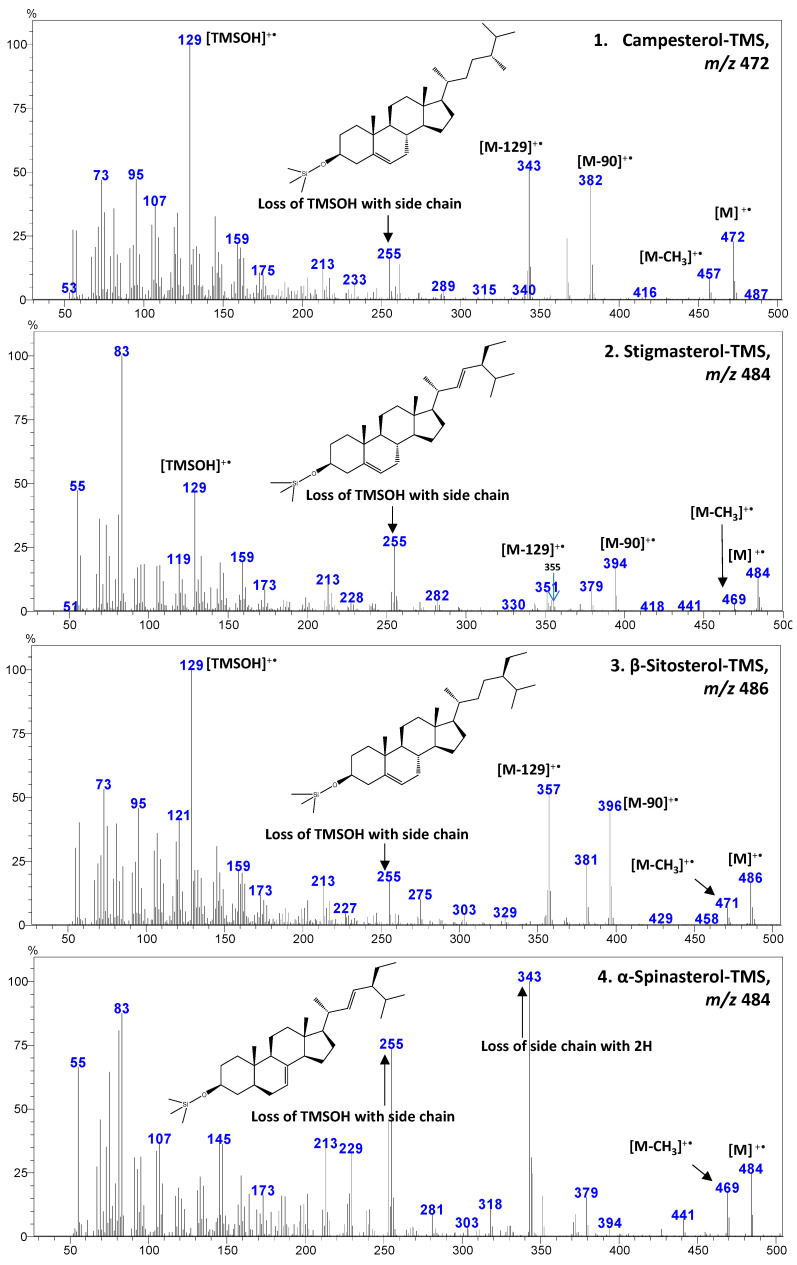
The gas chromatography (GC)-mass spectra of most abundant sterols identified in the present study from the seed spices of the Apiaceae family. The mass spectra of campesterol, stigmasterol, and β-sitosterol are from celery, while α-spinasterol is from Ajwain.

This study analyzed seed spices of the Apiaceae family, namely dill, celery, caraway, coriander, cumin, fennel, anise, and Ajwain, for sterol profile utilizing GC-MS after derivatization with trimethylsiloxy groups [TMS; −O-Si(CH_3_)_3_]. Studied spices showed a significant variation for the oil composition, recorded between 17.5 (anise) to 31.1% (Ajwain) (Figure 1). In the extracted oil, β-sitosterol (stigmast-5-en-3β-ol)-TMS and stigmasterol (stigmasta-5,22-dien-3β-ol)-TMS was found to be the most dominating, followed by an unidentified sterol 2 (*m*/*z* 486), and campesterol (ergost-5-en-3 β-ol)-TMS (Figure 2 and Appendix C). All the identified sterols were confirmed by retention time and mass spectrum with authentic standards. The mass spectrum of sterols identified from studied spices is shown in Figure 3. Similarly, the mass spectrum of three minor unidentified sterols are given in Appendix D. These sterols were quantified using the calibration curve of β-sitosterol. In the present study, the GC-MS method utilized for the identification of major sterols was found efficient, as the interfering non-sterols peaks (eluting with sterols) were eliminated (Figure 3 and Appendix C). However, with the interference of non-sterols compounds (peaks), GC-FID analysis may result in an error in the identification, as this technology is based only on retention time.

**Figure 4:**
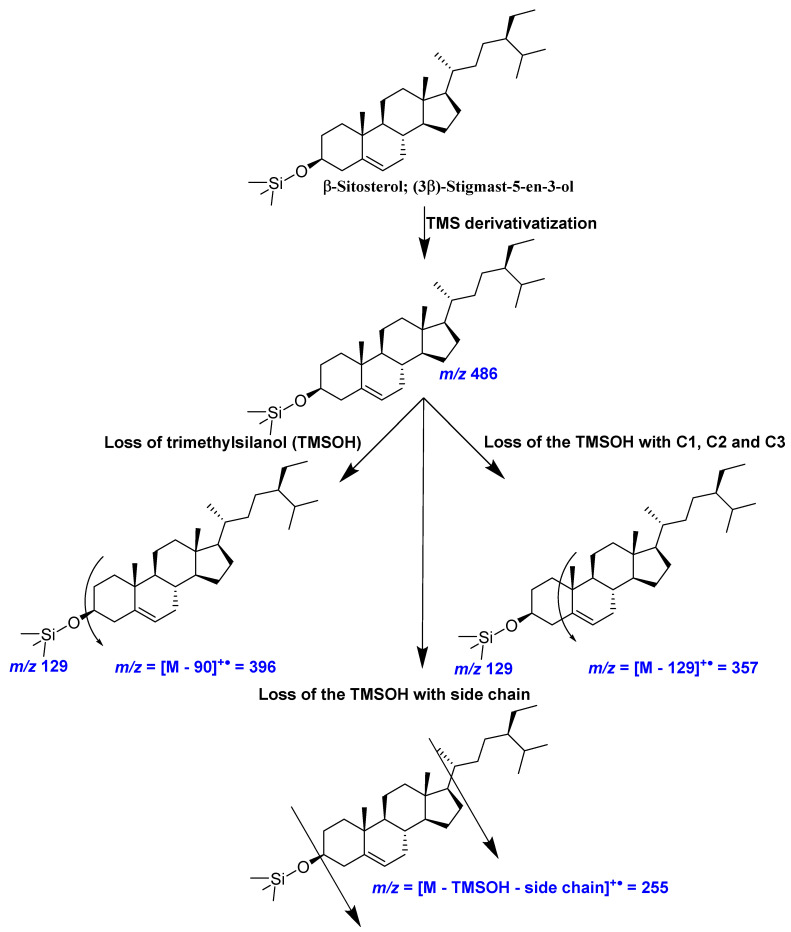
The mass spectrometric fragmentation pattern of β-sitosterol (TMS derivative) observed in the present study [26,27,28].

In the mass spectrometric identification of sterols, an ion at *m*/*z* [M-90]^+•^, for the loss of trimethylsilanol (TMSOH), and loss of the TMSOH with side chain is a common feature [26,27]. The Δ5-sterol TMS derivatives, such as campesterol, stigmasterol, and β-sitosterol give a particularly valuable and characteristic fragmentation involving loss of the TMS-group together with C-l, C-2, and C-3 of the sterol A-ring, yielding intense ions at *m*/*z* 129 for the TMS derivative-containing fragment and *m*/*z* [M-129]^+•^ for the remaining portion of the sterol (Figure 3 and Figure 4) [26]. In contrast, the Δ7-sterol TMS derivatives, such as α-spinasterol, produce characteristics ion [M-141] ^+•^ by losing side chain with two hydrogens (Figure 3) [28].

As evident from the identified sterols, the mass spectra were different among Δ5- (campesterol, stigmasterol, and β-sitosterol) and Δ7-sterols (α-spinasterol) (Figure 3). Moreover, Δ5,7-sterol (e.g., ergosterol) commonly occur in food, especially mushrooms [22], which may yield different mass spectra. In the present study, the unidentified sterol 1 showed [M]^+•^, [M-CH_3_]^+•^, [M-side chain with 2H]^+•^, and [TMSOH]^+•^ fragment ions (Appendix D). In the previous studies, a significant amount of Δ5-avenasterol (stigmasta-5,24-dien-3β-ol) has been recorded in the Apiaceae family seed spices, especially in coriander [16,21] and fennel [20]. In the present study, probably the unidentified Compound 1 is Δ5-avenasterol (which may yield the *m*/*z* of 484; TMS derivative); however, other major fragments, such as *m*/*z* 394 [M-90]^+•^ and *m*/*z* 355 [M-129]^+•^ were not observed, which were the common fragments of Δ5-sterol TMS (campesterol, stigmasterol, and β-sitosterol) recorded in the present study.

The unidentified Sterol 2 showed [M]^+•^ at surprisingly high intensity, with [M-CH_3_]^+•^ and [M-TMSOH-side chain with 2H]^+•^. In contrast, the unidentified Sterol 3 showed [M-TMSOH-side chain with 2H]^+•^ at high intensity, similar to α-spinasterol, suggesting that this is probably a Δ7-sterol TMS. However, their identity was not confirmed, as GC-MS alone is not adequate to elucidate the identity of unknown sterols [29].

### 3.2. Sterol Contents in Studied Spices

**Figure 5:**
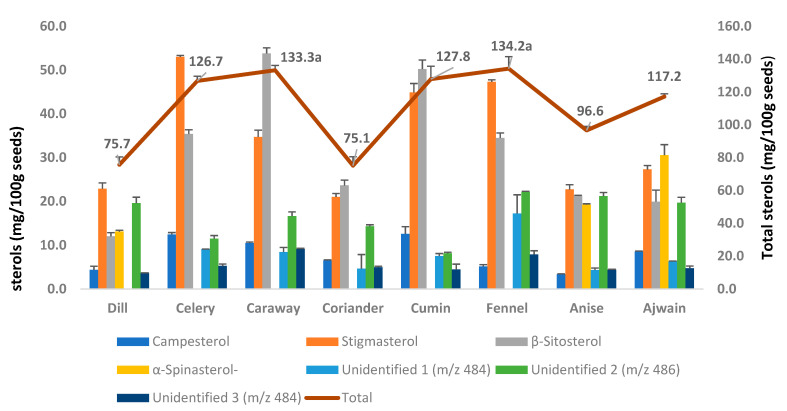
Phytosterol composition (mg/100 g seeds; dry weight basis) of studied Apiaceae family seed spices. Values are mean ± standard deviation of six replicates. The secondary axis represents the line graph. ^a^ The mean value is significantly highest among the studied spices (p < 0.05; Turkey HSD).

In the present study, the studied spices showed significantly different contents of individual and total sterols (*p* < 0.05; Turkey HSD). Sterols contents calculated as oil basis (mg/100 g of oil) and spice weight basis (mg/100 g spices) were given in Table 1 and Figure 5, respectively. Among the studied spices, the highest contents of total sterol were recorded in the oil extracted from caraway (602.2 mg/100 g oil), followed by anise (551.9 mg/100 g oil) and celery (546.2 mg/100 g oil) (Table 1). Interestingly, based on spice weight (Figure 5), fennel seeds also showed the similar highest number of total sterols (134.2 mg/100 g in fennel and 133.3 mg/100 g in caraway, dry weight basis; and were not statistically significant at *p* < 0.05, Turkey HSD), owing to the high contents of oil (25.9%) in fennel seeds with a considerable amount of sterols.

Among the sterol compounds, β-sitosterol was found to be the most dominating in the oil extracted from caraway (243.1 mg/100 g oil), cumin (196.3 mg/100 g oil), and coriander (121.7 mg/100 g oil), account for 40.4, 39.3, and 31.5% of total sterols, respectively. Interestingly, stigmasterol was most dominating in oil extracted from celery (228.6 mg/100 g oil), fennel (182.4 mg/100 g oil), and dill (109.3 mg/100 g oil), accounted for 41.8, 35.3, and 30.3% of total sterols, respectively.

In agreement with the present investigation, Balbino et al. [18] also recorded the dominance of campesterol, stigmasterol, and β-sitosterol in seed spices of the Apiaceae family, with stigmasterol as the dominating sterol in the oil extracted from fennel, and β-sitosterol in caraway and coriander seed oil. However, Balbino et al. [18] recorded significantly higher contents of β-sitosterol, stigmasterol, and α-spinasterol, and total sterol in anise seed oil than the contents recorded in the present investigation.

In the present study, the highest amount of α-spinasterol was recorded in anise seed oil (109 mg/100 g oil; 19.9% of total sterols), with a significant presence in dill (62.6 mg/100 g oil), and Ajwain seed oil (98.1 mg/100 g oil). In agreement with the present study, Balbino et al. [18] record a significant amount of α-spinasterol in anise seed oil, while it was not detected in previous studies [16,19].

A significant variation is recorded in the previous studies for the phytosterol profile of seed spices of the Apiaceae family (Table 2). Ramadan [21] recorded stigmasterol as the most dominating (29.8% of total sterol) in the coriander seeds oil, with dominating amount of β-sitosterol (28.2% total sterol), Δ5-avenasterol (23.8% of total sterol). However, in the present investigation, in coriander seed oil β-sitosterol was the most dominating (31.5% total sterol), followed by stigmasterol (28.0% of total sterol). In addition, Kozłowska [16] recorded the stigmasterol as the most dominating in coriander and caraway seeds oil, with a significant amount of Δ5-avenasterol.

Islam et al. [20] detected stigmasterol (57.6 mg/100 g seeds), β-sitosterol (48.8 mg/100 g seeds), and Δ5-avenasterol (40.9 mg/100 g seeds) as the dominating sterols in fennel seeds, with total sterol contents of 156.3 mg/100 g seeds, which is slightly higher than recorded in the present study. In the present study, campesterol accounted for 9.9% of total sterol; whereas, Hosseini [30] recorded 49.19% campesterol in cumin seeds oil. Heredity (cultivar/variety) [31,32], geographical conditions [33], and several other different biotic and abiotic factors [34] during cultivation and storage [35] are probably responsible for a significant variation in sterol contents and compositions among plants. Thus, the factors influencing sterols contents positively can be identified and utilized for enhancing the sterols contents in the food crops and diet.

In view of the health benefits of phytosterols in reducing the LDL-C levels [11,12,13] with antioxidant [14] and anti-inflammatory properties [15], consumption of phytosterol-rich seeds spices, such as celery, caraway, fennel, and anise seeds may improve the health benefits of the diet.

## 4. Conclusions

The GC-MS is an efficient method for sterol profiling, as the interfering non-sterols peaks (eluting with sterols) are eliminated. To the best of our knowledge, this is the first report of the GC-MS profile of phytosterols from dill, celery, and Ajwain seeds. The present study revealed that the phytosterol profile substantially varies among seed spices of the Apiaceae family. Among the studied spices, the oil extracted from caraway seeds showed the highest total phytosterols (602.2 mg/100 g of oil). In addition, oil extracted from caraway, fennel, and anise seeds were also recorded rich in phytosterols (517.3–551.9 mg/100 g of oil). Consumption of these spices may provide health benefits [11,12,13,14,15]. In the future, more detailed studies can help to uncover the identity of unidentified sterols.

## Figures and Tables

**Table 1:** Phytosterol composition of studied spices (mg/100 g of oil).

Peak No.	Sterol	RT (min)	*m*/*z* (M]^+•^	Dill	Celery	Caraway	Coriander	Cumin	Fennel	Anise	Ajwain
1.	Campesterol	27.141	472	20.8 ±4.0	53.6 ± 1.9 ^a^	47.4 ± 1.0	33.4 ± 0.8	49.2 ± 6.3 ^a^	19.8 ± 1.7	19.3 ± 0.5	27.5 ± 0.3
2.	Stigmasterol	27.991	484	109.3 ± 6.3	228.6 ± 1.3 ^a^	156.9 ± 7.1	108.2 ± 4.0	175.4 ± 8.0	182.4 ± 1.7	129.9 ± 6.2	87.7 ± 2.9
3.	β-Sitosterol	29.861	486	57.4 ± 4.0	152.7 ± 4.0	243.1 ± 5.9 ^a^	121.7 ± 6.2	196.3 ± 8.1	132.9 ± 4.5	121.7 ± 0.7	64.0 ± 8.5
4.	α-Spinasterol	29.904	484	62.6 ± 1.4	n.d.	n.d.	n.d.	n.d.	n.d.	109.9 ± 1.3 ^a^	98.1 ± 7.7
U1.	Unidentified 1 (*m*/*z* 484)	30.390	484	n.d.	39.2 ± 0.1	38.1 ± 4.7	23.9 ± 16.5	29.4 ± 2.2	66.4 ± 16.5 ^a^	24.7 ± 2.7	20.4 ± 0.1
U2	Unidentified 2 (*m*/*z* 486)	32.045	486	93.7 ± 6.4	49.5 ± 3.1	75.3±4.3	73.7 ± 1.8	31.9 ± 1.0	85.4 ± 0.6	121.2 ± 4.6 ^a^	63.3 ± 4.0
U3	Unidentified 3 (*m*/*z* 484)	32.951	484	17.5 ± 0.1	22.7 ± 1.9	41.4 ± 0.5 ^a^	25.5 ± 1.3	17.3 ± 4.9	30.4 ± 3.2	25.2 ± 0.6	15.2 ± 1.7
	Total			361.4 ± 22.4	546.2 ± 12.4	602.2 ± 12.9 ^a^	386.4 ± 28.1	499.5 ± 30.5	517.3 ± 28.3	551.9 ± 14.0	376.2 ± 5.5

Values are mean ± standard deviation from an average of three determinations. n.d.; not detected; RT: retention time. ^a^ The mean value is significantly highest among the studied spices (*p* < 0.05; Turkey HSD). Peak: the peak numbers correspond to those used in Figure 3 and Appendix C. The *m*/*z* values are from the TMS derivative of each sterol compound.

**Table 2:** Phytosterol composition of Apiaceae family seeds spices reported in previous studies.

Common Name	Oil Contents (%)	Campesterol	Stigmasterol	β-Sitosterol	α-Spinasterol	Δ7-Stigmasterol	Δ7-Avenasterol	Δ5-Avenasterol	Total Sterols	Analytical Technique Used	Reference
Caraway	8.14	38.8 ^a^	160.3 ^a^	240.2 ^a^	19.8 ^a^	29.3 ^a^	46.4 ^a^	n.d.	570.3 ^a^	GC-MS	[18]
	19.8	61.1 ^a^	79.1 ^a^	226.6 ^a^	n.d.	10.7 ^a^	8.8 ^a^	0.8 ^a^	405 ^a^	GC-MS	[30]
	18.9	31.7 ^a^	245.4 ^a^	244.9 ^a^	n.d.	n.d.	23.7 ^a^	87.1 ^a^	739.4 ^a^	GC-FID	[16]
Coriander	13.3	49.0 ^a^	109.6 ^a^	177.6 ^a^	18.3 ^a^	70.3 ^a^	24.3 ^a^	n.d.	468.8 ^a^	GC-MS	[18]
	22.1	36.6 ^a^	118.0 ^a^	100.9 ^a^	n.d.	n.d.	15.2 ^a^	54.4 ^a^	358.4 ^a^	GC-FID	[16]
	19.2	44.4 ^a^	136.9 ^a^	231.4 ^a^	n.d.	106.4 ^a^	26.9 ^a^	21.0 ^a^	629 ^a^	GC-MS	[17]
	-	50.8 ^a^	154.8 ^a^	146.4 ^a^	n.d.	n.d.	24.4 ^a^	123.5 a	518.6 ^a^	GC-FID	[21]
Cumin	26.8	214 ^a^	73.3 ^a^	104.1 ^a^	n.d.	14.0 ^a^	5.4 ^a^	11.5 ^a^	426 ^a^	GC-MS	[30]
Fennel	7.89	35.0 ^a^	172.5 ^a^	140.2 ^a^	19.7 ^a^	53.0 ^a^	30.9 ^a^	n.d.	492.4 ^a^	GC-MS	[18]
	20.1 ^c^	49 ^a^	223 ^a^	161 ^a^	n.d.	n.d.	18 ^a^	6 ^a^	464 ^a^	GC-FID	[19]
	-	9.0 ^b^	57.6 ^b^	48.8 ^b^	n.d.	n.d.	n.d.	40.9 ^b^	156.3 ^b^	GC-FID	[20]
Anise	5.98	67.7 ^a^	206.9 ^a^	211.6 ^a^	206.2 ^a^	140.4 ^a^	47.0 ^a^	n.d.	903.9 ^a^	GC-MS	[18]
	24.1 ^c^	119 ^a^	118 ^a^	238 ^a^	n.d.	n.d.	9 ^a^	3 ^a^	385 ^a^	GC-FID	[19]
	5.4	91.1 ^a^	67.9 ^a^	626.4 ^a^	n.d.	n.d.	n.d.	n.d.	849.9 ^a^	GC-FID	[16]

^a^ (mg/100 g oil); ^b^ (mg/100 g seeds); ^c^ highest oil yield utilizing Folch method. GC-FID: gas chromatography (GC)-flame ionization detection (FID); GC-MS: GC-mass spectrometry. Campesterol (ergost-5-en-3 β-ol; CAS 474-62-4), stigmasterol (Δ5-Stigmasterol; stigmasta-5,22-dien-3β-ol; CAS 83-48-7), β-sitosterol (stigmast-5-en-3β-ol; CAS 83-46-5), α-spinasterol ((22E)-stigmasta-7,22-dien-3ß-ol; CAS 481-18-5)), Δ7-stigmasterol (Stigmasta-5,7,22-trien-3-ol; CAS 481-19-6), Δ7-avenasterol (Stigmasta-7,24(28)-dien-3-ol; CAS 23290-26-8), and Δ5-avenasterol (stigmasta-5,24-dien-3β-ol; CAS 18472-36-1).

**Figure A1:**
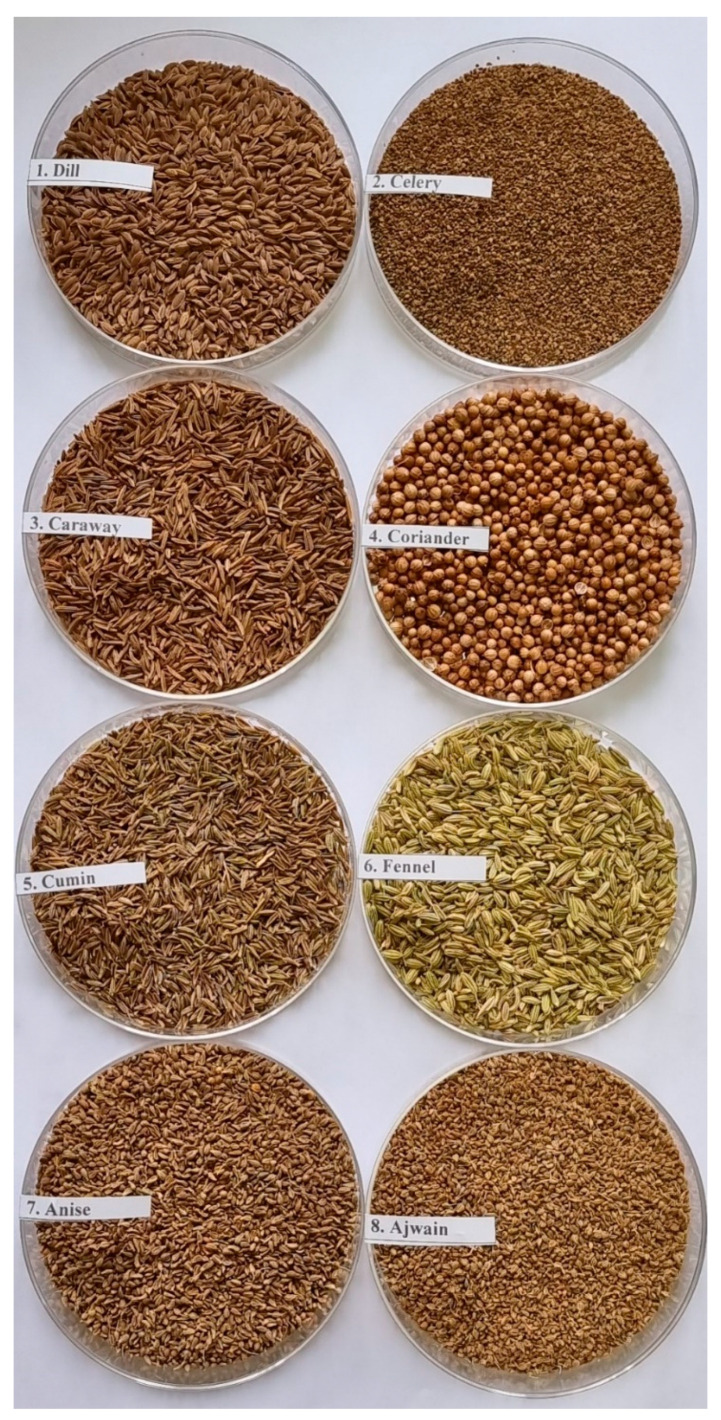
Apiaceae family spices used in the present study. Scale: the diameter of the Petri dish is 90 mm.

**Figure A2:**
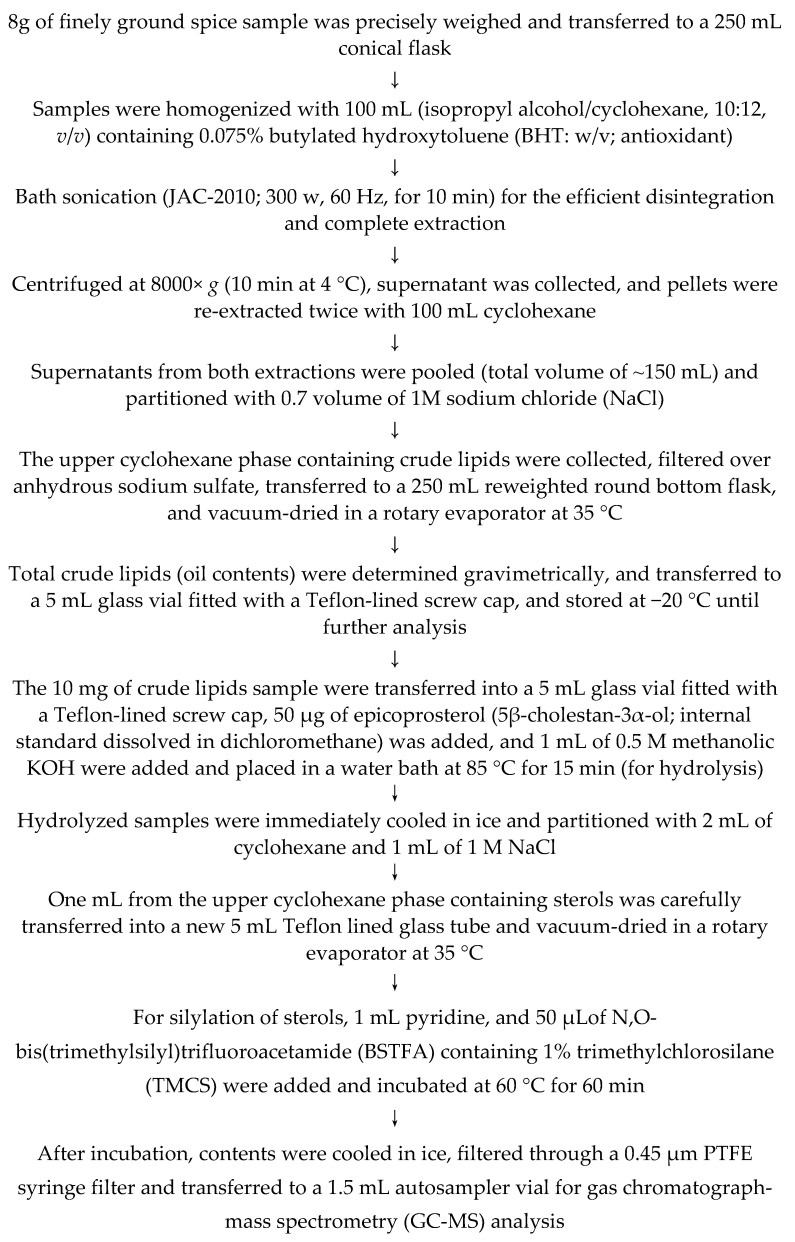
Outline of the method used for the extraction of crude lipids, hydrolysis, and silylation of sterols.

**Figure A3:**
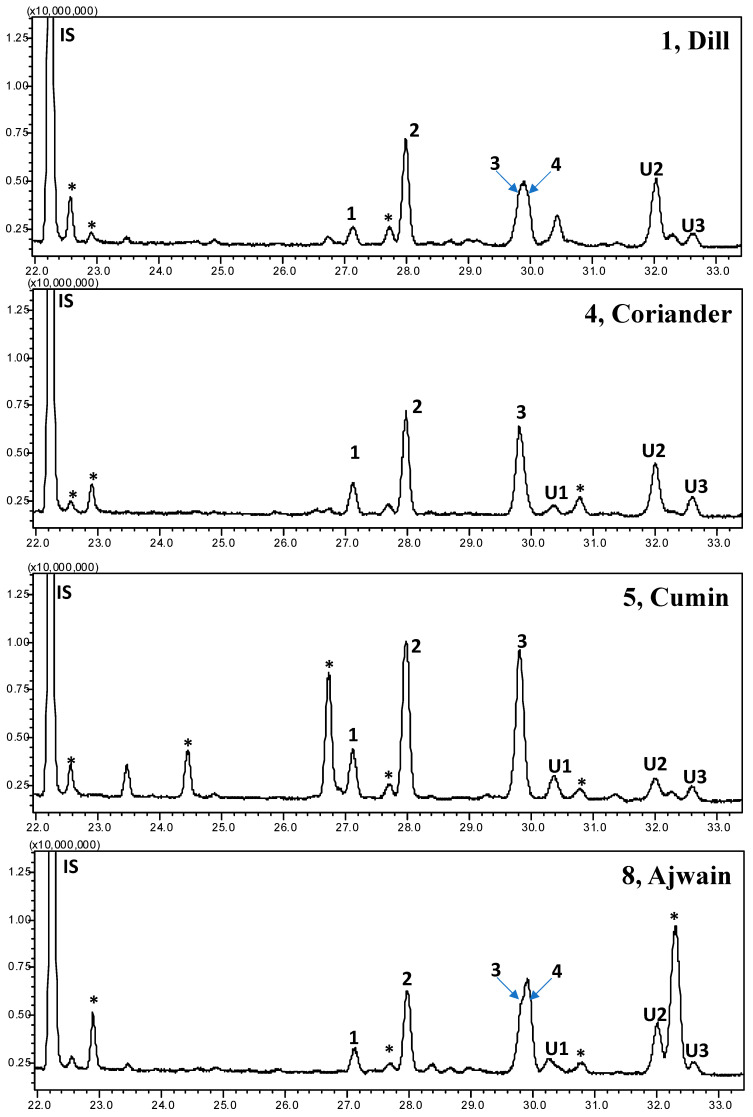
The gas chromatography (GC)- total ion chromatograms (TICs) of sterols identified in cumin, fennel, anise, and Ajwain seeds (seed oil). The peak numbers are as follows: (1) campesterol, (2) stigmasterol, (3) β-sitosterol, and (4) α-spinasterol. IS: internal standard (epicoprosterol; 5β-cholestan-3α-ol; CAS 516-92-7); U: unidentified; * non-sterols compounds.

**Figure A4:**
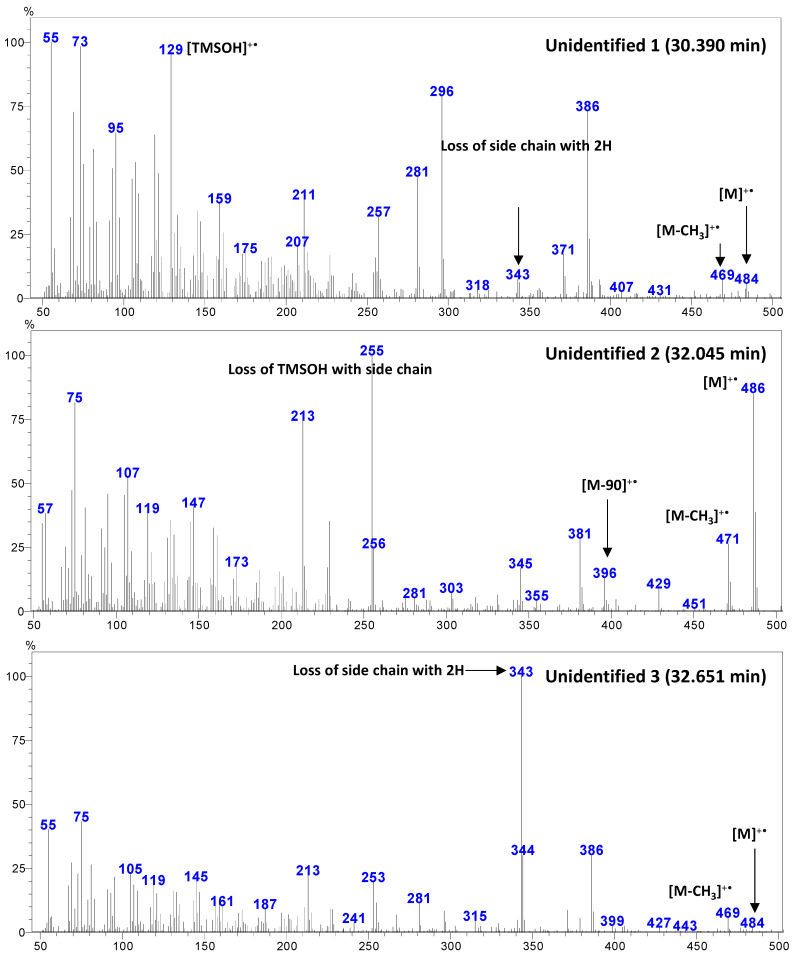
The gas chromatography (GC)-mass spectra of unidentified sterols (from the celery seeds).

## Data Availability

Data is contained within the article.
